# Use and misuse of ANOVA in in vitro biomaterials studies: A methodological analysis of the last ten years in the Journal of Clinical and Experimental Dentistry

**DOI:** 10.4317/jced.63758

**Published:** 2026-02-26

**Authors:** Marco Sánchez-Tito

**Affiliations:** 1 Research Group on Dental Biomaterials and Natural Products, Faculty of Health Sciences, Universidad Privada de Tacna, 23000 Tacna, Peru

## Abstract

**Background:**

This study aimed to estimate the frequency and characterize the nature of methodological errors related to the selection, application, and interpretation of ANOVA and related tests in in vitro biomaterials studies published in the Journal of Clinical and Experimental Dentistry (JCED) over the last ten years.

**Materials and Methods:**

A methodological review was conducted of original in vitro biomaterials articles published in JCED between 2016 and 2025. Studies reporting the use of ANOVA or derived procedures were included. Data were extracted on publication characteristics, statistical tests applied, verification of statistical assumptions, post hoc procedures, and statistical software. ANOVA use was classified as inappropriate when inconsistencies between reported and applied methods were identified, when assumption verification was inadequate or not specified, when post hoc procedures were inappropriate, or when statistical methods were incompletely reported.

**Results:**

A total of 345 in vitro biomaterials studies were included. Although ANOVA was widely used, complete verification of statistical assumptions based on model residuals was uncommon. The most frequent methodological errors associated with misused ANOVA were failure to specify the post hoc test used (50.0%) and inappropriate application of post hoc procedures (31.6%). Additional issues included incompatible post hoc tests following global analyses and discrepancies between methods and results.

**Conclusions:**

Despite its central role in in vitro dental research, ANOVA is frequently misused or inadequately reported in biomaterials studies. Strengthening statistical training and enforcing clearer editorial standards may substantially improve the validity, transparency, and reproducibility of experimental dental research.

## Introduction

Analysis of variance (ANOVA) is one of the most widely used statistical procedures in dental and biomedical research. Its widespread adoption is explained by its conceptual simplicity for comparing means across multiple groups and its routine integration into major statistical software packages. However, this accessibility has led to an uncritical use of ANOVA, even in situations where its fundamental assumptions are not met or where the interpretation of the results exceeds the analysis's scope (1 - 3). The inappropriate use of statistical tests in the biomedical literature has been extensively documented over the past few decades. Several methodological reviews have estimated that between 40% and 60% of scientific articles contain errors in the selection, application, or reporting of statistical methods, including ANOVA and its extensions (1 , 4 - 6). The most frequently reported errors include applying ANOVA to non-continuous variables, failing to verify assumptions of normality and homoscedasticity, omitting adjustments for multiple comparisons, and a reductionist interpretation of results based exclusively on p-values (3 - 7). From a conceptual perspective, one of the most common forms of ANOVA misuse is failing to recognize it as a global test. Kim described the persistence of incorrect practices, such as replacing ANOVA with multiple t-tests to compare more than two groups, a strategy that artificially inflates the type I error rate (3). Similarly, Abubakar et al., identified frequent errors, such as failing to specify the type of ANOVA used and omitting post hoc tests when required, thereby limiting analytical transparency and the reproducibility of findings (2 , 6). Within the dental research context, these methodological deficiencies have been repeatedly reported. Evaluations of dental journals have shown that a substantial proportion of published articles contain relevant statistical errors, mainly related to the use of parametric tests without verifying their assumptions and to incomplete descriptions of the analytical methods employed (8 - 10). Baccaglini et al., emphasized that many of these deficiencies originate at early stages of study design and cannot be corrected later through more complex statistical analyses (11). A particularly relevant issue in dental research is the intrinsic dependence among analytical units. Studies involving multiple teeth, surfaces, or repeated measurements within the same individual generate naturally correlated data, violating the independence assumption of ANOVA when the hierarchical structure of the data is not appropriately modeled (9 , 12). This issue has been described in both clinical and in vitro experimental studies (10 , 13). Repeated-measures designs represent another frequent scenario of ANOVA misuse. Repeated-measures ANOVA requires additional assumptions, particularly sphericity, whose omission may lead to biased inferences and inflation of the type I error rate (14 , 15). In this context, the use of more flexible alternative methods, such as mixed-effects models, has been recommended, particularly for longitudinal studies or datasets with missing observations (15 , 16). Recent evidence has shown that ignoring dependence among observations can lead to elevated false-positive rates, whereas mixed-effects models allow appropriate control of this bias and provide more robust inferences for data with complex hierarchical structures (16). Additionally, it has been reported that applying ANOVA to cumulative or time-dependent data may yield spurious statistical significance if the underlying dependency structure is not properly accounted for (7). Taken together, these observations highlight the need to evaluate not only the frequency of ANOVA use but also how it is selected, applied, and interpreted in contemporary dental research. Therefore, the aim of the present study was to estimate the frequency and characterize the nature of errors associated with the selection, application, and interpretation of ANOVA and its derived tests in in vitro biomaterials studies published in the Journal of Clinical and Experimental Dentistry (JCED) during the period 2016-2025.

## Material and Methods

A methodological review of original research articles was conducted. In vitro dental biomaterials studies were identified through a systematic search of the Journal of Clinical and Experimental Dentistry's official website (https://www.medicinaoral.com/odo/indice.htm). The search covered publications from issue 1 of volume 8 (2016) to issue 12 of volume 17 (2025), representing a 10-year publication period. All eligible articles were accessed online and downloaded in full for assessment. Articles were included if they reported the use of ANOVA or any related or derived statistical procedure. Identification of ANOVA use was performed by screening the Materials and Methods and Results sections for explicit mentions of ANOVA or its extensions, including textual descriptions, tables, and figures. For each included article, the following variables were extracted: year of publication, journal section, country of the principal author, reporting of the statistical test in the abstract, assessment of statistical assumptions, type of statistical test used, use of multiple-comparison procedures, and statistical software used. Data extraction was performed using a standardized data collection form. For the purposes of this study, the use of ANOVA was classified as inappropriate when one or more of the following criteria were identified: discrepancies between the statistical methods described in the Materials and Methods section and those reported in the Results; statements indicating that statistical assumptions were assessed without specifying the tests used; lack of specification of the test applied for multiple comparisons; inappropriate selection or application of post hoc tests; omission of the primary statistical test employed; and inappropriate selection of tests used to assess normality. Data extraction and methodological assessment were performed by a single investigator with formal training in biostatistics and research methodology. To ensure the reliability of the classification process and to minimize potential misclassification bias, an intra-rater agreement procedure was conducted. A random 10% subsample of the included articles was selected using the Excel random number generator and independently re-evaluated after a two-week interval. Agreement between the two assessments across all categorical variables was quantified using Cohen's kappa coefficient, which reached a value of 1.00, indicating perfect agreement. Data were analyzed using descriptive statistics, including absolute and relative frequency measures to characterize the variables of interest. Statistical processing was performed using Stata software, version 19 (StataCorp LLC, College Station, TX, USA). Graphical representations and additional data handling were conducted using R software (R Core Team, Vienna, Austria), through the RStudio integrated development environment (RStudio Team, Boston, MA, USA).

## Results

**Figure 1:**
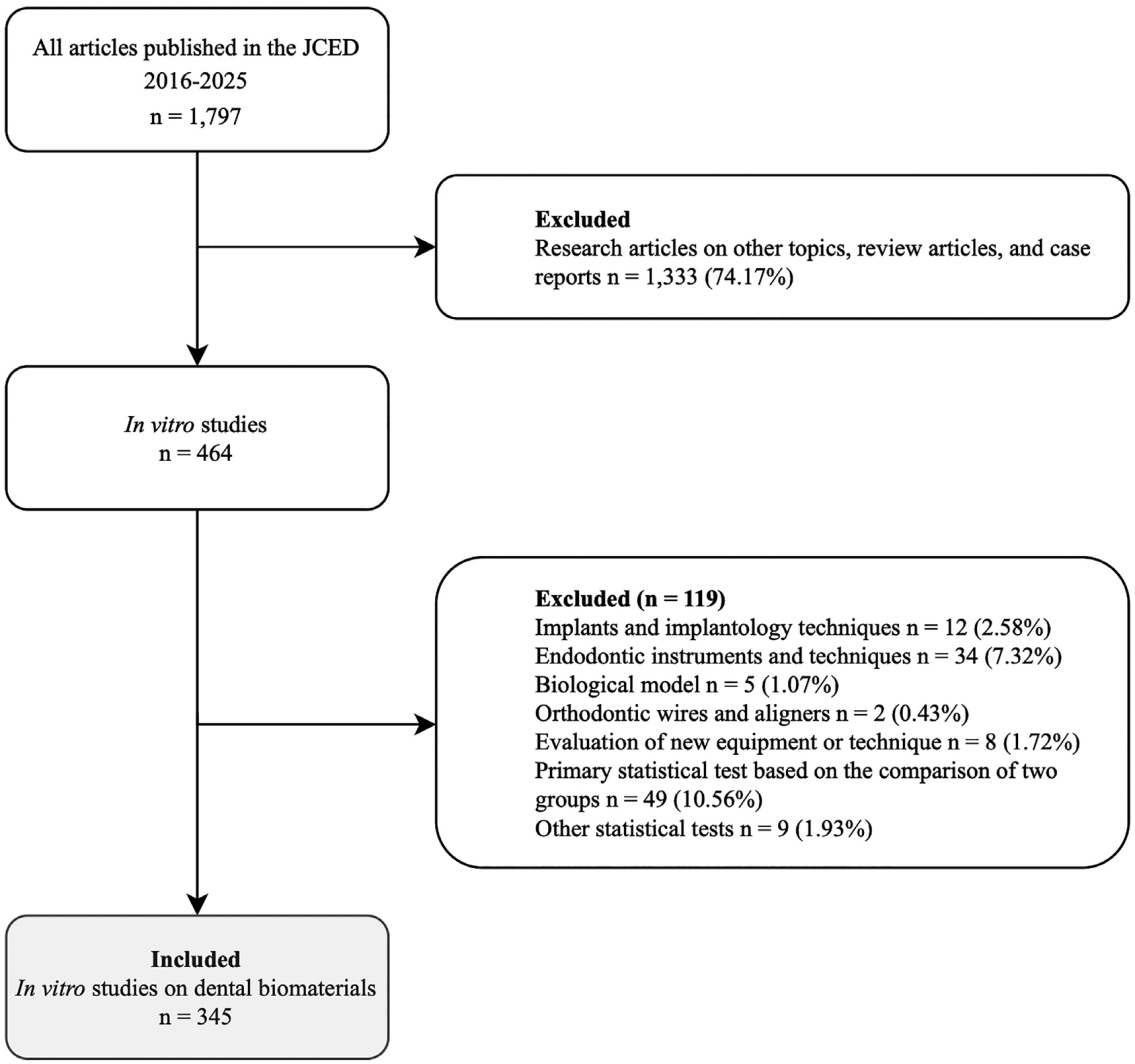
Flow chart of the study selection process.

The flow chart in Figure 1 summarizes the stepwise selection of articles included in the analysis.

[caption id="attachment_2053" align="alignnone" width="300"]1 Screenshot[/caption]

From a total of 1,797 articles published in the JCED between 2016 and 2025, 1,333 publications were excluded during the initial screening because they corresponded to non-in vitro studies, review articles, or case reports. Of the 464 in vitro studies identified, 119 were excluded for their thematic focus or for using statistical approaches not involving ANOVA. Finally, 345 in vitro studies on dental biomaterials met the eligibility criteria and were included in the methodological analysis. The distribution by publication year showed a heterogeneous pattern, with the highest frequencies observed in 2017 (17.39%) and 2025 (11.88%). Regarding journal sections, most articles were published in Operative Dentistry and Endodontics (40.29%), followed by Biomaterials and Bioengineering in Dentistry (17.10%) and Esthetic Dentistry (11.88%), while other sections contributed a minimal number of publications, (Table 1).

Table 1

**Figure 2:**
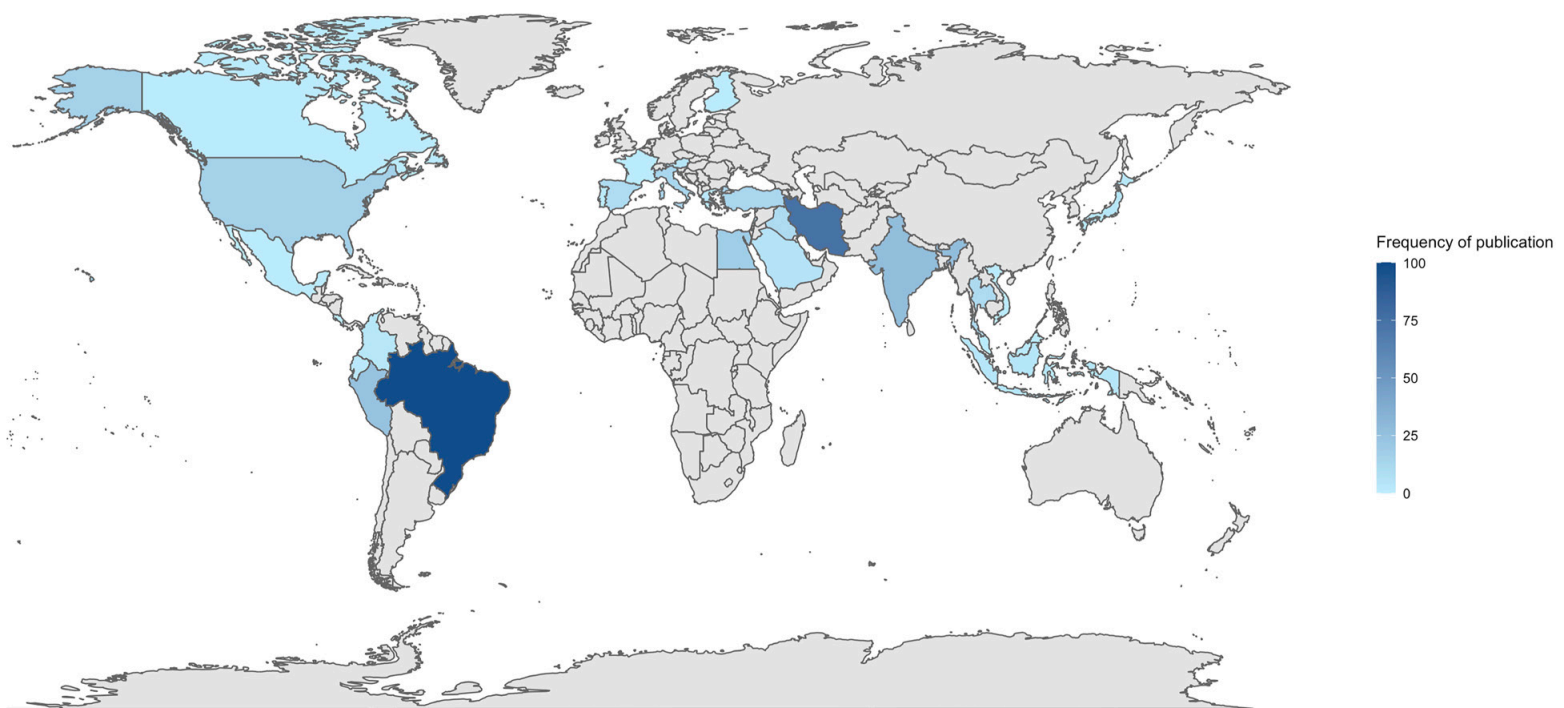
Worldwide distribution of ANOVA-based in vitro biomaterials research published in the Journal of Clinical and Experimental Dentistry (2016–2025).

Figure 2 shows a heterogeneous geographical distribution of publications, with a marked concentration in a small number of countries.

[caption id="attachment_2054" align="alignnone" width="300"]2 Screenshot[/caption]

**Figure 3:**
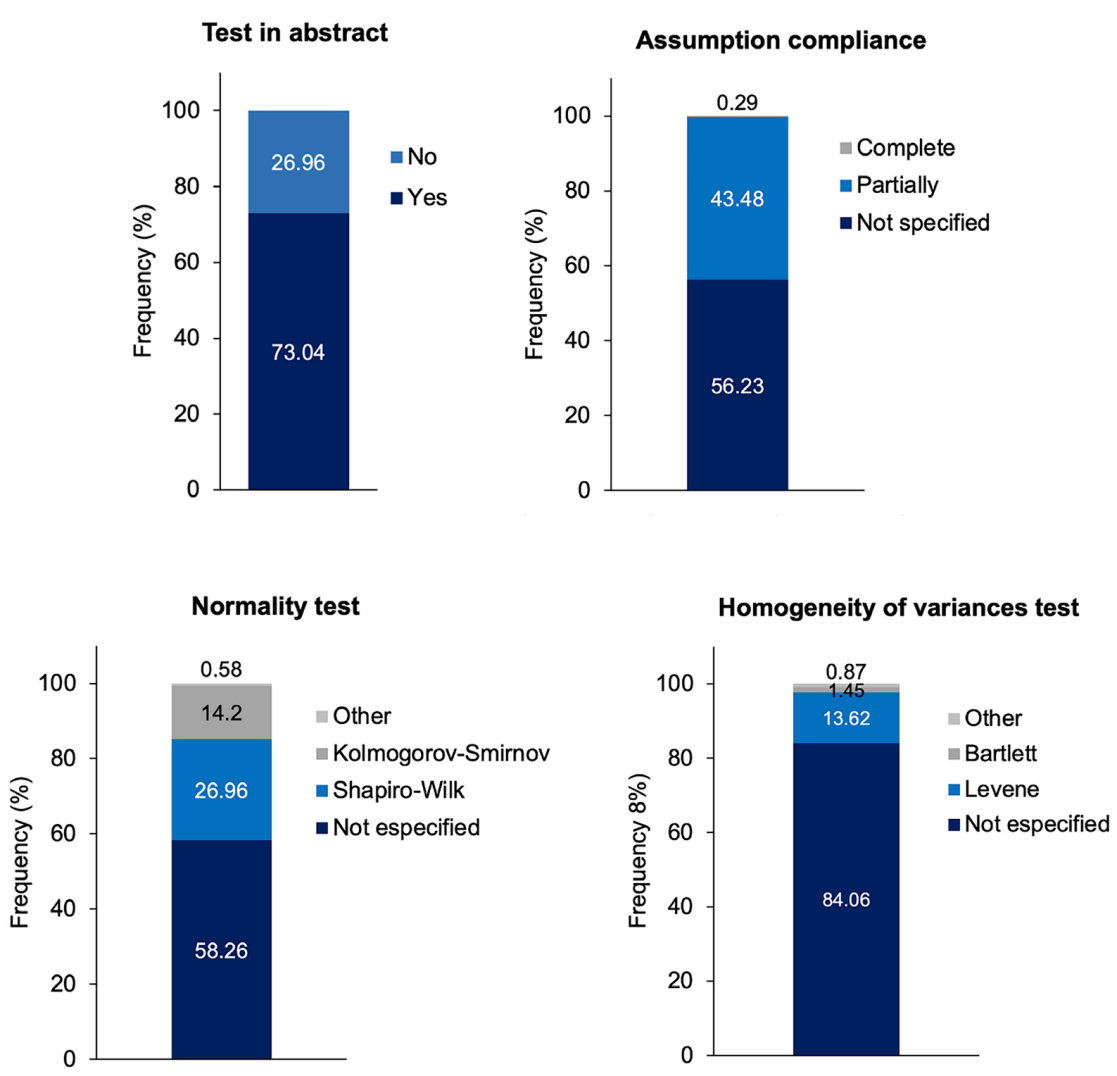
Reporting of statistical tests and assumption verification in included studies. The “complete” category corresponds to studies that evaluated statistical assumptions using model residuals or assumptions specific to the selected ANOVA model. The “partially” category includes studies that assessed only some assumptions or evaluated them based on raw data. The “not specified” category refers to studies in which no assessment of statistical assumptions was reported.

Brazil leads scientific output with 99 (28.70%), followed by Iran with 74 (21.45%), India with 27 (7.83%), and Peru with 24 (6.96%), positioning Latin America and Asia as the regions with the greatest overall contribution. In the Americas, in addition to Brazil and Peru, the United States stands out with 15 (4.35%), whereas in Europe the output is more dispersed, led by Italy and Turkey with 12 (3.48%) each, followed by Spain with 9 (2.61%). Figure 3 shows the reporting patterns of statistical tests and assumption verification among the included studies.

[caption id="attachment_2055" align="alignnone" width="300"]3 Screenshot[/caption]

**Figure 4:**
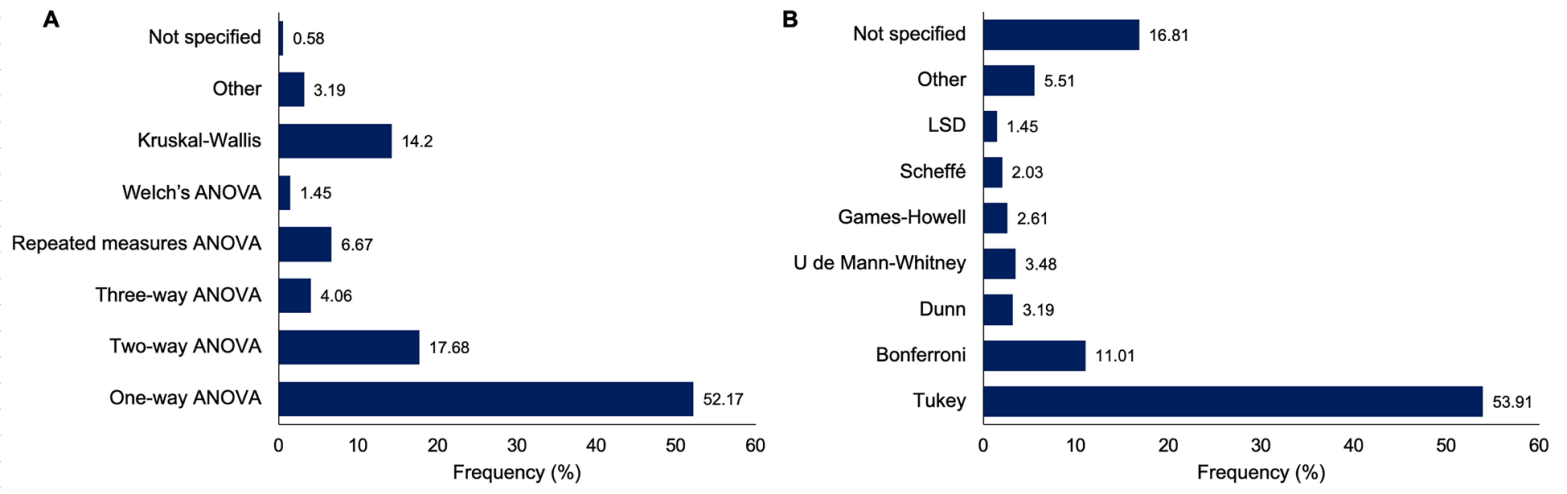
Distribution of main statistical tests and post hoc procedures used in the included studies.

Most articles reported the statistical test used in the abstract (73.04%). However, compliance with statistical assumptions was frequently incomplete or not specified, with only a small proportion of studies reporting a complete assessment. Regarding normality testing, more than half of the studies did not specify the test applied (58.26%), while the Shapiro-Wilk test was the most frequently reported among those that assessed normality. Similarly, homogeneity of variances was largely unreported (84.06%). When evaluating variance homogeneity, Levene's test was the most commonly used method (13.62%). Figure 4 shows the distribution of the main statistical tests (Panel A) and post hoc procedures (Panel B) reported in the included studies.

[caption id="attachment_2056" align="alignnone" width="300"]4 Screenshot[/caption]

One-way ANOVA was the most frequently used main analysis (52.17%), followed by two-way ANOVA (17.68%) and Kruskal-Wallis tests (14.20%). Other approaches, including repeated-measures ANOVA, three-way ANOVA, and Welch's ANOVA, were less frequently reported, while a small proportion of studies did not specify the statistical test used. Regarding post hoc analyses, Tukey's test was the most commonly reported procedure (53.91%), followed by Bonferroni (11.01%). Nonparametric post hoc tests, such as Dunn's test and the Mann-Whitney U test, were infrequently used. In addition, a considerable proportion of studies did not specify the post hoc method applied (16.81%), and other procedures, including Games-Howell and Scheffé tests, were reported only occasionally. Table 2 indicates that the most frequent methodological error associated with misused ANOVA was the failure to specify the test used for multiple comparisons (50.0%), followed by the inappropriate use of post hoc tests (31.6%), highlighting major deficiencies in the conduct and reporting of post hoc analyses.

Table 2

## Discussion

This study provides a methodological evaluation of the use and reporting of ANOVA and related procedures in in vitro biomaterials research published in the JCED over a ten-year period. The findings indicate that although ANOVA remains the predominant statistical tool, its application is characterized by persistent patterns of incomplete reporting and methodological inconsistencies that hinder the assessment of inferential validity. These results suggest that the main issue lies not in ANOVA itself, but in its uncritical application and limited analytical transparency. A key conceptual contribution of this study was the explicit distinction between partial and complete verification of statistical assumptions. Partial compliance included studies that reported assessing one or more classical assumptions without consistently specifying the level at which they were evaluated. In contrast, complete compliance was restricted to studies that evaluated assumptions based on the residuals, which represents the statistically appropriate approach in ANOVA and general linear models, as these assumptions pertain to the behavior of the error term rather than the marginal distribution of the observed data (17 , 18). Evaluating normality and homoscedasticity solely on raw data constitutes an incomplete and potentially misleading verification, particularly in multifactorial designs or studies with moderate sample sizes (19 , 20). In linear models, inference relies on the distribution and variance of the random error, making graphical and analytical inspection of residuals more informative and conceptually coherent than mechanical testing of original variables alone (18). Prior statistical and biomedical literature has consistently emphasized that reliance on marginal data normality may lead to suboptimal analytical decisions (1 , 3 , 11 , 21). Only one article adequately described the assessment of the residual-based assumption. The study by Tozo-Burgos et al., evaluated residual normality using the Shapiro-Wilk test and homoscedasticity using the Breusch-Pagan/Cook-Weisberg post-estimation test (22), an approach appropriate for two-way ANOVA since both assumptions are formulated at the level of the model error. In contrast, other studies reported normality tests without explicitly evaluating the homogeneity of the error term's variance (23 , 24). In factorial designs, main effects and interactions may induce heteroscedasticity across factor combinations (25). Detecting this pattern through residual-based assessments helps prevent inconsistent standard error estimates and reduces inflation of the type I error rate (26 , 27). This issue becomes particularly relevant in factorial and repeated-measures designs, common in in vitro biomaterials research, where multiple measurements are obtained from the same specimen. In such settings, additional assumptions such as sphericity are required, and failure to verify them may substantially inflate the type I error rate (15). Although a small number of studies applied robust approaches, including mixed or repeated-measures general linear models with appropriate corrections, these practices were infrequent (5). In the study by Neto et al., a three-way mixed-model ANOVA was used, with Box's M test applied to assess homogeneity of variance-covariance matrices and Mauchly's test with Greenhouse-Geisser correction to evaluate sphericity (28). This approach is methodologically appropriate for factorial repeated-measures designs, as it explicitly addresses key assumptions and reduces type I error inflation associated with sphericity violations and misspecified within-subject correlations, thereby supporting more valid inferences (29 , 30). Another key finding was the lack of coherence between the global statistical tests used and the post hoc procedures. In 12 studies that employed the nonparametric Kruskal-Wallis test, multiple comparisons were conducted using the Mann-Whitney U test. For instance, Da Silva et al., reported using the Kruskal-Wallis test followed by Mann-Whitney U tests with Bonferroni correction for pairwise comparisons (31). Similarly, Poggio et al., used a one-way ANOVA followed by pairwise t-tests (32). Both approaches are methodologically inappropriate, as neither the Mann-Whitney U test nor Student's t test constitutes a valid post hoc procedure after a multigroup global test. Repeated use of bivariate tests, even with simple multiplicity adjustments, disrupts the hierarchical logic of statistical inference and inflates the type I error rate, increasing the risk of spurious findings (13 , 33). When a global test is significant, multiple comparisons should instead be performed using procedures specifically designed for this purpose, such as Dunn, Conover-Iman, or Dwass-Steel-Critchlow-Fligner tests in the nonparametric setting, or Tukey, Bonferroni, or Games-Howell tests in the parametric context, which appropriately control the familywise error rate and preserve inferential consistency with the global test (33). Across multiple studies, methodologically incompatible combinations were identified, including the use of parametric post hoc tests following nonparametric global analyses, the performance of multiple comparisons without specifying the procedure used (34 - 39), and inconsistencies between the statistical methods reported in the Methods section and those applied in the Results. For example, Jonaidi-Jafari et al., reported the use of t tests and one-way ANOVA in the Methods, while the Results described the application of Mann-Whitney U and Kruskal-Wallis tests (40). These inconsistencies cannot be attributed solely to poor reporting, as in several cases the available information suggests a fundamentally incorrect application of inferential logic. Such errors have been widely documented in the biomedical literature and represent a major source of potentially biased conclusions (1 , 2 , 4). A recurrent issue was the conceptual confusion between post hoc testing and multiple-comparison corrections. For instance, Poggio et al. (41), and Albaheli et al. (42), reported the use of the Kruskal-Wallis test and stated that differences were evaluated "with Bonferroni correction" without specifying the post hoc procedure applied. This reporting is methodologically incorrect, as the Bonferroni adjustment only adjusts the significance threshold and does not specify the statistical method for pairwise comparisons (43). The absence of an explicitly stated post hoc test prevents verification of the coherence between the global rank-based test and subsequent comparisons, thereby compromising reproducibility and inferential validity. Regarding the assessment of normality and homogeneity of variances, a strong reliance on a limited set of tests, mainly Shapiro-Wilk and Levene, was observed, often without justification or complementary graphical inspection. Although widely accepted, their automatic use without considering sample size, design structure, or data characteristics limits their diagnostic value (3,20,44). When the homogeneity of variances assumption is violated, Welch's ANOVA has been proposed as a robust alternative to classical ANOVA, as it adjusts degrees of freedom to account for unequal variances and reduces type I error inflation (45). This approach was adopted in a subset of the studies published in JCED during the analyzed period. The consistent predominance of one-way ANOVA as the main analytical approach suggests a systematic oversimplification of potentially complex experimental designs. Many in vitro studies involve multiple experimental factors or repeated measurements, and reducing these designs to univariate analyses may mask important interaction effects and ignore within-unit dependence, thereby compromising inferential validity. In this context, mixed-effects models have been emphasized as a more flexible and conceptually appropriate alternative for analyzing data with hierarchical or correlated structures, particularly in experimental research (15,16,46). Notably, some studies applied more robust statistical approaches aligned with their objectives and data structure. Abdulhussein et al., used a general linear model repeated measures factorial ANOVA with Bonferroni post hoc testing to assess the combined effect of different lasers and fluoride on enamel demineralization resistance (47). Lopes et al., applied generalized linear models to evaluate color and microhardness changes, although assumption testing relied on Shapiro-Wilk and Levene tests applied to raw data (48). Jager et al., analyzed viscoelastic properties of flowable resin composites using mixed-effects models and repeated-measures ANOVA (49). Similarly, Sánchez-Tito et al., employed a two-factor MANOVA to evaluate the effects of staining beverages on multiple optical properties of provisional restorative materials (50). These approaches illustrate the importance of selecting statistical models that appropriately reflect the experimental design, accommodate correlated or multivariate outcomes, and align inferential methods with the underlying data structure, thereby improving the validity and interpretability of results. From an editorial perspective, the identified deficiencies appear to reflect recurrent patterns rather than isolated errors, largely driven by inadequate statistical reporting. This lack of clarity limits readers' and reviewers' ability to assess analytical adequacy and undermines reproducibility, underscoring the need for more explicit reporting of statistical methods to improve the methodological quality of experimental research (2 , 4). This study has inherent limitations, as it relies exclusively on the information reported in the published articles and cannot rule out that some appropriate analyses were performed but not adequately described. Nevertheless, this limitation underscores the critical importance of complete statistical reporting, particularly in in vitro biomaterials research, where multifactorial and repeated-measures designs are common and require careful application and documentation of ANOVA. In addition, the analysis was restricted to a single journal, and the aim was not to extrapolate the findings to the entire dental literature, but rather to provide an in-depth internal methodological assessment of the editorial and statistical practices of one specific journal. This journal-focused approach allows a more detailed and meaningful evaluation of reporting quality and analytical rigor and may serve as a model for similar audits of other dental journals, including the assessment of sample size planning and the use of alternative statistical models. In conclusion, although ANOVA remains a central analytical tool in in vitro dental research, its application and reporting frequently present methodological shortcomings. These deficiencies mainly involve incomplete assessment of statistical assumptions and inconsistencies between global tests and multiple-comparison procedures. Strengthening statistical training and implementing clearer, more explicit editorial guidelines could substantially enhance the validity, transparency, and reproducibility of experimental evidence in dentistry.

## Figures and Tables

**Table 1:** Table Distribution of included articles according to publication year and journal section.

Variable	Frequency (%)
Publication year	
2016	23 (6.67)
2017	60 (17.39)
2018	39 (11.30)
2019	33 (9.57)
2020	36 (10.43)
2021	30 (8.70)
2022	24 (6.96)
2023	25 (7.25)
2024	34 (9.86)
2025	41 (11.88)
Journal section	
Periodontology	4 (1.16)
Community and preventive dentistry	30 (8.70)
Esthetic dentistry	41 (11.88)
Biomaterials and bioengineering in dentistry	59 (17.10)
Operative dentistry and endodontics	139 (40.29)
Prosthetic dentistry	32 (9.86)
Orthodontics	31 (8.99)
Oral Medicine and pathology	4 (1.16)
Odontostomatology for the disabled or special patients	1 (0.29)
Oral surgery	2 (0.58)

1

**Table 2:** Table Frequency of methodological errors associated with misused ANOVA (n = 38)*.

Misuse	Frequency (%)
Inconsistency between the statistical methods described in the Materials and Methods section and those reported in the Results	1(2.63)
Reports that statistical assumptions were assessed without specifying the tests performed	1(2.63)
Does not specify the test used for multiple comparisons	19(50.00)
Inappropriate use of post hoc tests	12(31.58)
Does not specify the primary statistical test used	1(2.63)
Inappropriate use of tests to assess normality	4(10.53)

* The table shows the frequency of methodological errors other than the lack of complete residual-based assumption assessment, which was not fulfilled by most of the analyzed articles (n = 343).

## Data Availability

The datasets used and/or analyzed during the current study are available from the corresponding author.
